# Serum total cholesterol and risk of cardiovascular and non-cardiovascular mortality in old age: a population-based study

**DOI:** 10.1186/s12877-017-0685-z

**Published:** 2017-12-28

**Authors:** Yajun Liang, Davide Liborio Vetrano, Chengxuan Qiu

**Affiliations:** 1 Aging Research Center, Department of Neurobiology, Care Sciences and Society, Karolinska Institutet-Stockholm University, Stockholm, Sweden; 2 Department of Public Health Sciences, Karolinska Institutet, Widerströmska huset, 171 77 Stockholm, Sweden; 3 Department of Geriatrics, Catholic University of Rome, Rome, Italy; 4 Department of Neurology, Shandong Provincial Hospital Affiliated to Shandong University, Jinan, Shandong China

**Keywords:** Cardiovascular disease, Elderly, Mortality, Population study, Total cholesterol

## Abstract

**Background:**

Whether the suggested inverse association between total cholesterol and mortality in old age varies according to cause of death and use of cholesterol medications remains to be elucidated. The aim of this study was to assess the associations of total cholesterol with cardiovascular and non-cardiovascular mortality in old age, and to explore whether their associations vary by use of cholesterol-lowering medications.

**Methods:**

The study participants included 3090 older adults (age ≥ 60 years, 63.7% women) from a population-based cohort study, i.e., the Swedish National study on Aging and Care in Kungsholmen, Stockholm. At baseline (2001–2004), data on demographic factors, lifestyles, cardiovascular risk factors, use of medications, global cognitive function, mobility limitation, and apolipoprotein E genotype were collected through interviews, clinical examinations, laboratory tests as well as from the Swedish national patient register. Vital statistics data (e.g., date and causes of death) till December 31, 2011 for all participants were derived from Swedish cause of death register. Data were analyzed using Cox proportional hazards model for all-cause mortality and Fine-Gray competing risks regression model for cause-specific mortality controlling for multiple potential confounders.

**Results:**

During 23,196 person-years of follow-up (median per person, 7.5 years), 1059 (34.3%) participants died. Compared to normal total cholesterol (<5.18 mmol/l), borderline-high (5.18–6.21 mmol/l) and high (≥6.22 mmol/l) total cholesterol were associated with a decreased risk of all-cause mortality, with the multiple-adjusted hazard ratio (95% confidence interval, CI) of 0.71 (0.61–0.83) and 0.68 (0.57–0.80), respectively (*P* for trend <0.001). The competing risk regression models revealed that the reduced all-cause mortality associated with high total cholesterol (≥6.22 mmol/l)) was mainly due to the reduced risk of non-cardiovascular mortality (hazard ratio = 0.67, 95% CI = 0.51–0.88). These associations were statistically evident only among individuals without use of cholesterol-lowering medications.

**Conclusions:**

The inverse association between high total cholesterol and reduced all-cause mortality in older adults is primarily due to non-cardiovascular mortality, especially among those who are not treated with cholesterol-lowering medications.

## Background

It has been well studied that high levels of total cholesterol increase the risk of mortality among young and middle-aged people, however, there might be an attenuation of the association between total cholesterol and mortality with increase of age [1, 2]. Instead, low levels of cholesterol may be an independent predictor of poor survival among older adults, especially in the frailest groups [3–5]. However, there is scarce evidence from population-based studies on the association between total cholesterol and cause-specific mortality in older adults. The Rotterdam Study found that higher total cholesterol was associated with a lower risk of non-cardiovascular mortality in older adults (age ≥ 65 years), and the strength of the inverse association was increased with every decade increase in age [6].

Furthermore, whether the treatments of hypercholesterolemia (e.g., statins) are beneficial among very old people (e.g., age ≥ 80 years) remains a controversial dilemma. For instance, a meta-analysis of randomized placebo-controlled clinical trials suggested that statin therapy reduced all-cause mortality and cardiovascular mortality in older adults aged 60 years and older [7]. However, another review of observational studies and randomized controlled trials suggested that use of statins may even increase the all-cause mortality among very old people without cardiovascular diseases [8]. In addition, it remains uncertain whether use of cholesterol-lowering medications (e.g., statins) might modify the association of total cholesterol with all-cause and cause-specific mortality among older adults.

In this study, we seek to assess the longitudinal associations between total cholesterol and all-cause or cause-specific mortality in old age, and to explore whether their associations vary by use of cholesterol-lowering medications.

## Methods

### Study design and participants

Data were derived from the prospective population-based Swedish National study on Aging and Care in Kungsholmen (SNAC-K) (www.snac-k.se). The study participants of SNAC-K project were randomly selected from a central area of Stockholm. The eligible participants were those who aged ≥60 years and lived either at home or in institutions at the time of enrollment. At baseline (March 2001–June 2004), 3363 persons (73.3% of all eligible) participated in the SNAC-K study [9, 10]. Of these, 273 persons were excluded owing to missing data on total cholesterol, leaving 3090 participants for the current analyses.

### Data collection and definitions

At baseline, data on demographic factors (e.g., age, sex and education), lifestyles (e.g., smoking, alcohol consumption and physical activity), cardiovascular risk factors (e.g., obesity, hypertension and diabetes), use of medications, global cognitive function (e.g., Mini-Mental State Examination [MMSE]), mobility limitation (e.g., walking speed), and apolipoprotein E (APOE) genotype were collected through interviews, clinical examinations, laboratory tests, as well as from the Swedish national patient register.

Cognitive impairment was defined based on MMSE score and the dementia diagnosis according to the Diagnostic and Statistical Manual of Mental Disorders, 4th Edition [11]. Those who had a MMSE score ≤ 26 or had a diagnosed dementia were considered to be cognitively impaired. Walking speed (meters per second, m/s) was assessed by trained nurses based on the self-selected speed of walking 6 or 2.4 m, as previously described [12]. In brief, the length of the walk test was determined by asking the participants how fast they normally walk. Subjects who rated themselves as fast or normal walkers did the longer walk test and those who self-rated as slow or very slow walkers did the shorter walk test. At home visits, the shorter walk test was always conducted due to space restrictions. Walking speed reflects the speed (meters per second, m/s) from whichever walk test that was performed by the participant. Subjects who were unable to walk without personal support received the worst possible score, i.e. 0 m/s. Those who had a walking speed <0.8 m/s were defined as having mobility limitation [13].

Non-fasting peripheral blood samples were taken, and total cholesterol was measured. Total cholesterol levels were divided into three categories according to the Third Report of the National Cholesterol Education Program [14]: <5.18 mmol/l as normal, 5.18–6.21 mmol/l as borderline high, and ≥6.22 mmol/l as high total cholesterol. Self-report use of cholesterol-lowering medications was collected, and the drug prescriptions and containers were used to further verify the use of medications. Cholesterol-lowering agents were identified as code C10 according to the Anatomical Therapeutic Chemical classification system.

Survival status and causes of deaths until December 31, 2011 for all participants were ascertained from the Swedish cause of death register. Causes of death were classified according to the International Classification of Diseases, Tenth Revision (ICD-10) and categorized into cardiovascular diseases (ICD-10 codes: I00-I99) and non-cardiovascular diseases (due to all other reasons) [9].

### Statistical analysis

Baseline characteristics between alive participants and those who died during follow-up were compared using univariate analysis of variance for continuous variable and binary logistic regression or multinomial logistic regression for categorical variables after controlling for age. Cox proportional hazards model was performed to estimate the association between total cholesterol and all-cause mortality, from which hazard ratio (HR) and 95% confidence interval (CI) of all-cause mortality were estimated. The log-log plots of survival curves were used to verify the proportional hazards assumption. Fine-Gray competing risks regression models were employed to assess the associations between total cholesterol and cause-specific (cardiovascular and non-cardiovascular) mortality [15]. Furthermore, we assessed the interaction between total cholesterol and use of cholesterol-lowering medications on the risk of mortality. If a statistical interaction was detected, we performed the analyses stratified by use of cholesterol-lowering medications. Various factors were controlled as covariates in the models, e.g., age, sex, education, current smoking, heavy alcohol drinking, physical inactivity, obesity, hypertension, diabetes, APOE ε4 allele, cognitive impairment, mobility limitation, and if possible, for use of cholesterol-lowering drugs.

IBM SPSS 22 for Windows (IBM SPSS Inc., Chicago, Illinois, USA) and SAS version 9.4 (SAS Institute Inc., Cary, NC, USA) were used for all analyses.

## Results

The mean age of the 3090 participants was 73.3 (standard deviation, 10.4) years and 63.7% were women.

During 23,196 person-years of follow-up (range, 0.1–10.8 years; median per person, 7.5 years), 1059 (34.3%) participants died. Compared with those who were still alive during follow-up, those who died during follow-up were older (*P* < 0.001), less likely to have university education or above (*P* = 0.020), less likely to be former smokers (*P* = 0.022) or current smokers (*P* < 0.001), more likely to be physically inactive (*P* < 0.001), had a lower level of total cholesterol (*P* < 0.001), had a higher prevalence of diabetes (*P* < 0.001), cognitive impairment (*P* < 0.001), and mobility limitation (*P* < 0.001), and less likely to be an APOE ε4 carrier (*P* = 0.046) after controlling for age. The two groups did not differ significantly in the proportion of sex and in the prevalence of heavy alcohol drinking, obesity, hypertension, and use of cholesterol-lowering drugs (Table 1).

**Table 1:** Baseline characteristics of study participants by survival status at follow-up

	Total	Survival status at follow-up		
Characteristics ^a^		Alive	Died	*P* ^b^
No. of subjects	3090	2031	1059	
Age (years), mean (SD)	73.3 (10.4)	69.0 (8.5)	81.5 (8.5)	<0.001
Female, n (%)	1969 (63.7)	1298 (63.9)	671 (63.4)	0.764
Education, n (%)				
Elementary or middle school	505 (16.4)	223 (11.0)	282 (26.7)	–
High school	1536 (49.8)	978 (48.2)	558 (52.8)	0.003
University or above	1046 (33.9)	830 (40.9)	216 (20.5)	0.020
Smoking, n (%)				
Never	1442 (47.0)	908 (45.0)	534 (50.9)	–
Former smoking	1178 (38.4)	812 (40.2)	366 (34.9)	0.022
Current smoking	447 (14.6)	298 (14.8)	149 (14.2)	<0.001
Heavy alcohol drinking, n (%)	495 (16.1)	376 (18.6)	119 (11.4)	0.406
Physical inactivity, n (%)	955 (30.9)	409 (20.1)	546 (51.6)	<0.001
Obesity, n (%)	374 (12.1)	278 (13.7)	96 (9.1)	0.539
Hypertension, n (%)	2318 (75.3)	1448 (71.4)	870 (82.8)	0.887
Diabetes, n (%)	293 (9.5)	146 (7.2)	147 (14.0)	<0.001
Total cholesterol (mmol/l), mean (SD)	6.0 (1.1)	6.1 (1.1)	5.8 (1.2)	<0.001
Use of cholesterol-lowering drugs, n (%)	381 (12.4)	270 (13.3)	111 (10.6)	0.784
Cognitive impairment, n (%)	432 (14.0)	95 (4.7)	337 (31.9)	<0.001
Mobility limitation, n (%)	826 (26.9)	231 (11.4)	595 (57.2)	<0.001
APOE ε4, n (%)	835 (29.1)	569 (29.2)	266 (28.7)	0.046

*APOE ε4* apolipoprotein E ε4

^a^Data were missing in 3 subjects (3 dead) for education, 23 (10 dead) for smoking, 19 (11 dead) for alcohol consumption, 11 (8 dead) for hypertension, 12 (6 dead) for diabetes, 8 (7 dead) for use of cholesterol-lowering medication, 5 (4 dead) for cognitive impairment, 23 (19 dead) for walking speed, and 216 (133 dead) for ApoE4. These factors were considered as covariates in subsequent analyses, in which a dummy variable for each of these factors was created to represent the group of subjects with missing value

^b^ *P*-value is for the test of difference between those died and alive controlling for age

In the total sample, compared to normal level of total cholesterol (<5.18 mmol/l), borderline high (5.18–6.21 mmol/l) and high (≥6.22 mmol/l) levels of total cholesterol were significantly associated with decreased all-cause mortality (*P* for trend <0.001), with the multivariable-adjusted HRs (95% CIs) being 0.71 (0.61–0.83) and 0.68 (0.57–0.80), respectively (Table 2). Furthermore, the competing risk regression models revealed that the reduced risk of all-cause mortality associated with high total cholesterol was mainly due to the reduced risk of non-cardiovascular mortality (*P* for trend = 0.004); there was no significant association between levels of total cholesterol and risk of cardiovascular mortality (Table 2).

**Table 2:** Association of total cholesterol with all-cause, cardiovascular, and non-cardiovascular mortality

Total		All-cause mortality			Cardiovascular mortality			Non-cardiovascular mortality		
cholesterol (mmol/l)	No. of subjects	No. of deaths	Mortality rate (per 1000 person-years)	Hazard ratio (95% CI)^a^	No. of deaths	Mortality rate (per 1000 person-years)	Hazard ratio (95% CI)^b^	No. of deaths	Mortality rate (per 1000 person-years)	Hazard ratio (95% CI)^b^
Total sample										
< 5.18	687	325	71.8	1.00 (Ref.)	205	45.3	1.00 (Ref.)	120	26.5	1.00 (Ref.)
5.18–6.21	1207	394	43.2	0.71 (0.61–0.83)	236	25.9	0.82 (0.66–1.02)	158	17.3	0.85 (0.66–1.09)
≥ 6.22	1196	340	35.6	0.68 (0.57–0.80)	221	23.2	0.98 (0.77–1.23)	119	12.5	0.67 (0.51–0.88)
*P* for trend				<0.001^a^			0.882^b^			0.004^b^
No use of cholesterol-lowering medications										
< 5.18	495	259	84.7	1.00 (Ref.)	162	53.0	1.00 (Ref.)	97	31.7	1.00 (Ref.)
5.18–6.21	1066	358	44.7	0.70 (0.59–0.82)	213	26.6	0.79 (0.63–1.00)	145	18.1	0.88 (0.67–1.16)
≥ 6.22	1140	324	35.5	0.65 (0.54–0.77)	209	22.9	0.91 (0.72–1.17)	115	12.6	0.69 (0.51–0.92)
*P* for trend				<0.001^a^			0.573^b^			0.009^b^
Use of cholesterol-lowering medications										
< 5.18	189	64	44.0	1.00 (Ref.)	42	28.9	1.00 (Ref.)	22	15.1	1.00 (Ref.)
5.18–6.21	138	33	29.6	0.76 (0.49–1.19)	22	19.7	0.84 (0.47–1.50)	11	9.9	0.75 (0.35–1.61)
≥ 6.22	54	14	33.2	1.03 (0.56–1.90)	11	26.0	1.47 (0.77–2.81)	3	7.1	0.52 (0.14–1.98)
*P* for trend				0.961^a^			0.561^b^			0.244^b^

^a^Hazard ratios (95% confidence intervals, CIs) were derived from Cox regression models that were controlled for age, sex, education, current smoking, heavy alcohol drinking, physical inactivity, obesity, hypertension, diabetes, APOE ε4 allele, cognitive impairment, mobility limitation, and if applicable, for use of cholesterol-lowering drugs

^b^Hazard ratios (95% CIs) were derived from the competing risks model that was controlled for all variables mentioned in the above footnote

**Fig. 1:**
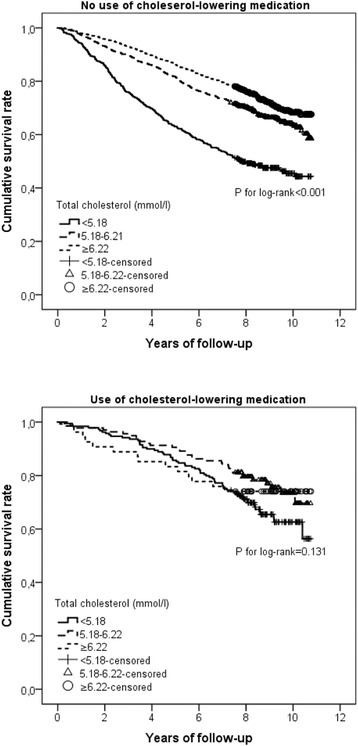
Kaplan-Meier survival curves for different levels of total cholesterol by use of cholesterol-lowering medications

In addition, we detected a statistical interaction between total cholesterol and use of cholesterol-lowering medications on the risk of mortality. Further analysis stratified by use of cholesterol-lowering drugs suggested that the reduced risk of all-cause or non-cardiovascular mortality associated with high total cholesterol was statistically evident only among individuals who did not use cholesterol-lowering medications (Table 2 and Fig. 1).

## Discussion

Our population-based cohort study of older adults showed that compared to normal total cholesterol, higher levels of total cholesterol were significantly associated with decreased all-cause mortality. Furthermore, the competing risk regression models revealed that the reduced risk of all-cause mortality associated with high total cholesterol was mainly due to the reduced risk of non-cardiovascular mortality. Finally, the observed inverse associations between total cholesterol and risk of all-cause mortality and non-cardiovascular mortality were present only among individuals who did not use cholesterol-lowering medications.

The finding of an association between higher levels of cholesterol and lower risk of all-cause and non-cardiovascular mortality in older adults was consistent with the previous studies, which showed that high levels of total cholesterol were associated with prolonged survival in elderly people, largely owing to the lower mortality from cancer and infections [5, 16]. Furthermore, the reduced risk of all-cause or non-cardiovascular mortality associated with high total cholesterol was evident only among individuals who were not treated with cholesterol-lowering medications. This was in line with the finding from a study of patients with stroke showing that the increased risk of mortality associated with low cholesterol was present only among those who did not use statins prior to the onset of stroke [4].

However, the biological and pathophysiological pathways linking serum cholesterol to mortality in the aging process are poorly understood. Lipids and lipoproteins may play a protective role by modulating the inflammation markers, such as C-reactive protein, cytokines, tumor necrosis factor and interleukin 6 [17–19]. Thus, low levels of cholesterol may cause elderly people to be vulnerable to inflammatory processes. On the other hand, low total cholesterol may be a marker of poor nutritional status, frailty and clinical complexity of chronic health conditions in older people [20–22], which may in turn increase the risk of mortality [23, 24]. In our study, when we assessed the association between total cholesterol and mortality, we controlled for multiple chronic health conditions (e.g., obesity, hypertension, and diabetes) and markers of frailty (e.g., cognitive impairment and mobility limitation). Further research is warranted to better understand the mechanisms underlying the potential survival benefits resulting from high cholesterol among older adults.

The tolerability of cholesterol-lowering agents in the elderly people (e.g., age ≥ 75 years) has become a concern, because the potential harm of intensive treatment of high cholesterol might be greater in older adults than that in young and middle-aged people [25]. The current guidelines do recommend that the treatment and control of blood cholesterol among elderly people should be with additional cautions [26, 27]. For instance, the 2011 guidelines from the European Society of Cardiology and European Atherosclerosis Society recommended that clinician judgement is urged for the treatment of high blood cholesterol in elderly people [26]. The 2013 guidelines from the American College of Cardiology and the American Heart Association recommended a lower intensity of statin therapy for the elderly people than for young and middle aged people to reduce the risk of atherosclerotic cardiovascular disease [27].

The present study has many strengths, e.g., population-based study design, long follow-up, and appropriate statistical analysis (i.e., competing risks models). However, this study also has limitations. First, we only focused on total cholesterol measured and we were not able to examine the associations between various components of lipid profiles (e.g., low-density lipoprotein cholesterol, high-density lipoprotein cholesterol and triglyceride) and mortality due to lack of data in our project. Second, cardiovascular risk reduction might at least partly rely on types, dosages, period and adherence of cholesterol-lowering therapy [28], but we could not address these issues because of lack of the relevant information. Third, other potential residual confounding (e.g., nutritional status), which are related to both total cholesterol and mortality, cannot be completely ruled out. Finally, we only controlled for potential confounders measured at baseline without taking into account the changes of these factors during follow-up, which might also affect mortality.

## Conclusion

Higher levels of total cholesterol are associated with lower risk of all-cause mortality, especially non-cardiovascular mortality, among older people; the association is evident mainly among individuals who are not treated with cholesterol-lowering medications. Our study adds to the emerging evidence that the associations of total cholesterol with all-cause mortality may vary with age, cause of death and medical treatment. Caution might be needed for therapeutic control of blood cholesterol among elderly people in the perspective of long-term risk for cardiovascular and non-cardiovascular mortality.

## Abbreviations

APOE: Apolipoprotein E gene
CI: Confidence interval
HR: Hazard ratio
ICD-10: International classification of diseases, tenth revision
MMSE: Mini-mental state examination
SNAC-K: Swedish National study on Aging and Care in Kungsholmen
